# Long noncoding RNA MALAT1 polymorphism predicts MACCEs in patients with myocardial infarction

**DOI:** 10.1186/s12872-022-02590-0

**Published:** 2022-04-07

**Authors:** Tong Zhang, Jun-Yi Luo, Fen Liu, Xue-He Zhang, Fan Luo, Yi-Ning Yang, Xiao-Mei Li

**Affiliations:** 1 Department of Cardiology, The First Affiliated Hospital of Xinjiang Medical University, 137 Liyushan South Road, Urumqi, 830054 Xinjiang China; 2 Xinjiang Key Laboratory of Cardiovascular Disease Research, Urumqi, 830054 Xinjiang China; 3 People’s Hospital of Xinjiang Uygur Autonomous Region, 91 Tianchi Road, Urumqi, 830054 Xinjiang China

**Keywords:** lncRNA MALAT1, Myocardial infarction, Single nucleotide polymorphism, MACCE, rs3200401

## Abstract

**Background:**

Metastasis-associated lung adenocarcinoma transcript 1 (MALAT1) participates in the occurrence and development of cardiovascular and cerebrovascular diseases such as stroke and coronary heart disease by regulating inflammatory reactions, programmed cell death, and other pathological processes. Previous studies revealed that the *MALAT1* gene polymorphism was associated with cardiac and cerebrovascular diseases. However, the prognostic role of the *MALAT1* polymorphism in major adverse cardiac and cerebrovascular events (MACCEs) remains unknown. Therefore, this study intends to explore the association between the *MALAT1* rs3200401 polymorphism and MACCEs.

**Method:**

We enrolled 617 myocardial infarction (MI) patients and 1125 control participants who attended the First Affiliated Hospital of Xinjiang Medical University from January 2010 to 2018. SNPscan™ typing assays were used to detect the *MALAT1* rs3200401 genotype. During the follow-up, MACCEs were recorded. Kaplan–Meier curves and univariate and multivariate Cox survival analyses were used to explore the correlation between *MALAT1* gene polymorphisms and the occurrence of MACCEs.

**Results:**

Among the total participants and MI patients, the frequencies of the T allele (total Participants 19.5% vs. 15.3%, *P* = 0.047, MI patients 20.7% vs. 14.1%, *P* = 0.014) and CT + TT genotypes (total Participants 37.4% vs. 28.1%, *P* = 0.013, MI patients 39.5% vs. 25.8%, *P* = 0.003) were significantly higher in subjects with MACCEs than in subjects without MACCEs. However, in control participants, the frequencies of the T allele (16.6% vs. 16.0%, *P* = 0.860) and CT + TT genotypes (31.4% vs. 29.3%, *P* = 0.760) were not higher in subjects with MACCEs than in subjects without MACCEs. In addition, among the total participants and MI patients, the Kaplan–Meier curve analysis indicated that the subjects with rs3200401 CT + TT genotypes had a higher incidence of MACCEs than CC genotype carriers (*P* = 0.015, *P* = 0.001). Nevertheless, similar results were not observed in the control participants (*P* = 0.790). Multivariate Cox regression indicated that compared with patients with the CC genotype, patients with CT + TT genotypes had a 1.554-fold increase in MACCE risk (hazard ratio: 1.554, 95% confidence interval: 1.060–2.277, *P* = 0.024).

**Conclusions:**

The *MALAT1* rs3200401 CT + TT genotypes could be a risk factor for MACCEs in MI patients, suggesting that the *MALAT1* gene may become a biomarker for poor prognosis in MI patients.

## Introduction

Currently, with the aging of Chinese society and the development of urbanization, cardiac and cerebrovascular diseases, such as stroke, hypertension, and coronary heart disease, have become the leading killer diseases in China. Myocardial infarction (MI) is the main cause of mortality and disability among cardiac and cerebrovascular diseases. The death rate of MI increased by 3 times from 2010 to 2018 [1]. MI is necrosis of ischemic myocardium caused by occlusive thrombosis in the coronary artery [2]. For MI patients, early reperfusion therapies include fibrinolysis, percutaneous coronary intervention, and coronary artery bypass graft surgery. The long-term treatment strategy is mainly secondary prevention, including antithrombotic therapy, β-blocker treatment, lipid-lowering therapy, and lifestyle interventions [3]. However, current treatments cannot completely prevent major adverse cardiac and cerebrovascular events (MACCEs).

Long noncoding RNAs (lncRNAs) are usually transcripts of at least 200 base pairs in length that do not code for proteins. In the previous literature, researchers found that lncRNAs were involved in the incidence and development of MI. For example, lncRNA ZFAS1 could regulate sarcoplasmic reticulum Ca^2+^-ATPase 2a protein to impair cardiac contractile function in MI [4]. LncRNA CAIF suppresses cardiac autophagy and attenuates MI by binding p53 [5]. In addition, overexpression of lncRNA DACH1 could block cardiac repair and regeneration post-MI [6].

Metastasis-associated lung adenocarcinoma transcript-1 (MALAT1) is an 8779 bp transcript located at 11q13. The *MALAT1* gene was first discovered in lung adenocarcinomas and reported to be a crucial factor in several human diseases, such as tumors [7], cardiovascular disease [8], and pulmonary arterial hypertension [9]. In the study of myocardial infarction, *MALAT1* can regulate the programmed death of cardiomyocytes [10] and mediate cardiac fibrosis of cardiac fibroblasts [11], thereby promoting myocardial infarction. Hu et al. found that after 2 weeks of ligation of the left anterior descending coronary artery, the *MALAT1* gene expression level increased 3 times in the infarct zone of the mouse heart, while the miR-320 expression level decreased, and a dual-luciferase reporter confirmed that lncRNA MALAT1 may directly target miR-320, thereby increasing cardiomyocyte apoptosis [12].

In previous studies, we investigated the relationship between the *MALAT1* gene polymorphism and the occurrence of the acute coronary syndrome, including myocardial infarction [13]. However, the prognostic role of the *MALAT1* polymorphism in MACCE has not been discovered. This study intended to explore the association of the *MALAT1* gene rs3200401 polymorphism with MACCEs among control participants and MI patients.

## Materials and methods

### Study population

This was a case–control study to investigate the relationship between *MALAT1* gene polymorphisms and MACCE susceptibility in Xinjiang Province. All participants were recruited at the First Affiliated Hospital of Xinjiang Medical University from 2010 to 2018.

The diagnosis of MI was made according to the guidelines [14]. All MI participants underwent arteriography to verify coronary artery stenosis (> 50% reduction luminal diameter). The Gensini score was calculated according to the angiography result to evaluate the extent of coronary stenosis [15].

We also recruited control participants who had no history of cardiovascular diseases and no signs of ischemic heart disease. The exclusion criteria for all participants were valvular heart disease, congenital heart disease, nonischemic cardiopulmonary disease, or acute and chronic inflammatory diseases.

### Biochemical analysis

Peripheral venous blood samples (5 mL) were collected in EDTA-containing tubes from all participants following overnight fasting for biochemical assays. Subsequently, the samples were centrifuged at 5000 rpm for 5 min to separate plasma from blood cells at 4 °C. Some plasma samples were sent to the Central Laboratory of the First Affiliated Hospital of Xinjiang Medical University for biochemical assays, including glucose, total cholesterol (TC), triglycerides (TG), low-density lipoprotein-cholesterol (LDL-C), high-density lipoprotein-cholesterol (HDL-C), apolipoprotein A (ApoA), apolipoprotein B (ApoB), and lipoprotein(a) (Lp(a)), using a commercially available automated platform. Blood cells were kept at − 80 °C separately for further analysis [16].

### Definition of cardiovascular risk factors

Body mass index (BMI) was calculated by dividing body weight (in kilograms) by height in meters squared. Smokers were regarded as people who regularly smoked for over 6 months. Hypertension was defined as systolic blood pressure (SBP) above 140 mmHg or diastolic blood pressure (DBP) above 90 mmHg, having previously been diagnosed by a physician or taking antihypertensive drugs [17]. A person who had abnormal fasting blood glucose or abnormal glucose tolerance was defined as a diabetes patient according to the Chinese guidelines for type 2 diabetes mellitus [18].

### Genetic polymorphism selection and genotyping

We utilized a commercial whole blood genome extraction kit to extract DNA from peripheral blood leukocytes according to the manufacturer’s instructions (Tiangen Biotech, China). We used SNPscan™ typing assays to detect polymorphisms of the *MALAT1* gene. The probes for the identification of primer sequences were 5′-TGCATTTACTTGCCAACAGAACAGAAAG-3′ and 5′-TGCATTTACTTGCCAACAGAACAGAGAA-3′. The universal primer was 5′-ACCTGAAGTCAAGACAACTGCATTC-3′.

### Follow-up and description of endpoints

In the evaluation of long-term clinical outcomes, MACCEs were considered as the endpoints, including cardiac and noncardiac death, nonfatal acute myocardial infarction, unplanned revascularization (new percutaneous coronary intervention or bypass cardiac surgery), malignant arrhythmia, development of congestive heart failure, and stroke. Cardiovascular events were defined according to the European Society of Cardiology (ESC) guidelines and the Standardized Definitions for Cardiovascular and Stroke End Point Events in Clinical Trials of the Clinical Data Interchange Standards Consortium (CDISC) [3, 19]. Participants were followed up every 6 months after discharge through telephone communications or face-to-face interviews with the patients or their family members by a trained research cardiologist using a structured questionnaire. Finally, 518 patients (29.7%) were lost to follow-up.

### Statistical analysis

Data analysis was performed using Statistical Package for Social Sciences-SPSS (version 22.0, SPSS Institute, Chicago, IL, USA). Continuous variables are expressed as the mean ± standard deviation (SD) or median (quartile), and the difference between the two groups was detected by independent-sample t-test or nonparametric rank test. Categorical variables are presented as n (%), and the difference between the two groups was detected by the chi-square test or Fisher’s exact test. For the analysis of the risk for MACCEs, univariate/multivariate Cox regression analyses were calculated for the influence of the risk factors on MACCEs. The results of the Cox models are presented as the hazard ratio (HR) and 95% confidence interval (CI). Kaplan–Meier analysis was used to assess the associations between participants’ survival rate and the *MALAT1* polymorphism. A *P* value < 0.05 was deemed statistically significant.

## Results

### General characteristics of participants

Table 1 shows the general characteristics of the enrolled participants. A total of 495 MI patients (mean age 57.75 ± 12.58 years and 82.2% men) and 729 control participants (mean age 54.60 ± 10.03 years and 48.2% men) were recruited in the present study.

**Table 1:** Clinical and demographic characteristics among the total participants, control participants and MI patients

	Total (n = 1224)			Controls (n = 729)			MI patients (n = 495)		
	nonMACCE (n = 1050)	MACCE (n = 174)	*P* value	nonMACCE (n = 678)	MACCE (n = 51)	*P* value	nonMACCE (n = 372)	MACCE (n = 123)	*P* value
Male, n (%)	641 (61.1%)	118 (67.9%)	0.434	334 (49.3%)	18 (35.3%)	0.288	307 (82.5%)	100 (81.3%)	0.938
Age (years)	55.05 ± 10.92	60.86 ± 11.82	< 0.001	54.53 ± 9.96	59.76 ± 9.80	0.006	56.53 ± 12.38	63.01 ± 12.47	< 0.001
BMI (kg/m2)	25.83 ± 3.56	25.54 ± 3.30	0.419	26.25 ± 3.91	25.88 ± 3.51	0.703	25.63 ± 3.26	25.29 ± 3.25	0.488
Hypertension, n (%)	450 (42.9%)	88 (50.6%)	0.246	300 (44.3%)	24 (47.1%)	0.796	105 (28.2%)	64 (52.0%)	0.002
Smoker, n (%)	411 (39.1%)	65 (37.4%)	0.816	195 (28.8%)	10 (19.6%)	0.338	216 (58.1%)	55 (44.7%)	0.180
Drinker, n (%)	324 (30.9%)	47 (27.0%)	0.493	172 (25.4%)	8 (15.7%)	0.241	152 (40.9%)	39 (31.7%)	0.231
Diabetes, n (%)	156 (14.9%)	42 (24.1%)	0.014	72 (10.6%)	8 (15.7%)	0.361	84 (22.6%)	34 (27.6%)	0.411
Glucose (mmol/L)	5.45 (4.71, 7.47)	7.16 (5.27, 9.36)	< 0.001	4.94 (4.53, 5.64)	4.76 (4.29, 5.29)	0.086	7.58 (6.35, 10.30)	8.01 (6.74, 10.58)	0.027
TG (mmol/L)	1.55 (1.05, 2.25)	4.28 (3.56, 5.04)	0.014	1.51 (1.02, 2.13)	1.25 (0.955, 1.705)	0.139	1.60 (1.08, 2.48)	1.42 (0.865, 2.10)	0.189
TC (mmol/L)	4.34 ± 1.07	4.37 ± 1.21	0.909	4.24 ± 0.94	4.07 ± 0.91	0.355	4.66 ± 1.18	4.71 ± 1.37	0.785
HDL-C (mmol/L)	1.08 ± 0.29	1.02 ± 0.27	0.014	1.12 ± 0.32	1.12 ± 0.28	0.992	1.04 ± 0.23	1.00 ± 0.24	0.257
LDL-C (mmol/L)	2.79 ± 0.86	2.86 ± 0.98	0.331	2.69 ± 0.8	2.59 ± 0.74	0.494	3.02 ± 0.89	3.04 ± 1.18	0.895
ApoA (mmol/L)	1.22 ± 0.31	1.16 ± 0.27	0.018	1.23 ± 0.26	1.13 ± 0.24	0.032	1.20 ± 0.44	1.19 ± 0.29	0.795
ApoB (mmol/L)	0.87 ± 0.30	0.88 ± 0.33	0.792	0.85 ± 0.25	0.82 ± 0.26	0.469	0.94 ± 0.40	0.94 ± 0.41	0.987
Lp (a) (mmol/L)	144.80 (88.13, 253.53)	157.99 (99.41, 265.90)	0.622	137.79 (86.61, 232.21)	137.48 (97.28, 224.08)	0.961	165.00 (91.51, 278.47)	176.68 (105.91, 301.97)	0.763
SBP (mmHg)	123.43 ± 17.08	122.84 ± 20.03	0.715	125.39 ± 16.39	124.12 ± 19.62	0.666	118.93 ± 16.77	121.54 ± 20.66	0.260
DBP (mmHg)	122.84 ± 20.03	76.29 ± 11.78	0.292	76.71 ± 11.02	75.74 ± 13.70	0.663	75.26 ± 12.55	73.85 ± 13.24	0.398

The continuous variables are defined as the mean ± SD or median (quartile). Categorical variables are expressed as percentages

The *P* value of the continuous variables was calculated by the independent samples t-test. The *P* value of the categorical variables was calculated by the Chi-square test

*BMI* body mass index, *SBP* systolic blood pressure, *DBP* diastolic blood pressure, *TG* triglyceride, *TC* total cholesterol, *HDL-C* high-density lipoprotein-cholesterol, *LDL-C* low-density lipoprotein-cholesterol, *ApoA* apolipoprotein A, *ApoB* apolipoprotein B, *Lp(a)* lipoprotein(a)

The median follow-up time was 60 months (IQR: 36, 82). During the follow-up period, 12.2% (174/1224) participants experienced MACCEs, 24.8% (123/495) MACCEs occurred in MI patients, and 7.0% (51/729) MACCEs occurred in control participants. The proportion of MACCEs in MI patients was higher than that in control participants (*P* < 0.001). During the follow-up period, of the 174 patients who had MACCEs, 19 patients had cardiac deaths, 22 patients had noncardiovascular death, 19 patients had cerebrovascular events, and 110 patients had cardiac events, including nonfatal acute myocardial infarction, unplanned revascularization (new percutaneous coronary intervention or bypass cardiac surgery), malignant arrhythmia, and the development of congestive heart failure.

Among all participants, compared with the nonMACCE group, participants in the MACCE group were older (60.86 ± 11.82 vs. 55.05 ± 10.92, *P* < 0.001) and had a higher proportion of diabetes patients (24.1% vs. 14.9%, *P* = 0.014), higher levels of admission blood glucose (7.16 (5.27, 9.36) vs. 5.45 (4.71, 7.47), *P* < 0.001), higher levels of TC (4.28 (3.56, 5.04) vs. 1.55 (1.05, 2.25), *P* = 0.014), and lower levels of HDL-C (1.02 ± 0.27 vs. 1.08 ± 0.29, *P* = 0.014) and lower levels of ApoA (1.16 ± 0.27 vs. 1.22 ± 0.31, *P* = 0.018).

In MI patients, compared with the nonMACCE group, patients in the MACCE group were older (63.01 ± 12.47 vs. 56.53 ± 12.38, *P* < 0.001) and had a higher proportion of hypertension patients (52.0% vs. 28.2%, *P* = 0.002) and higher levels of admission blood glucose [8.01 (6.74, 10.58) vs. 7.58 (6.35, 10.30), *P* = 0.027].

For the control participants, compared with the nonMACCE group, subjects in the MACCE group were also older (59.76 ± 9.80 vs. 54.53 ± 9.96, *P* < 0.001) and had lower levels of ApoA (1.13 ± 0.24 vs. 1.23 ± 0.26, *P* = 0.032).

### Distribution of *MALAT1* gene polymorphism

Among the total participants, the MACCE group had higher frequencies of the rs3200401 T allele and CT + TT genotypes than the nonMACCE group (T allele, 19.5% vs. 15.3%, *P* = 0.047, CT + TT genotypes, 37.4% vs. 28.1%, *P* = 0.013, Table 2).

**Table 2:** Distribution of MALAT1 gene rs3200401 among total participants, control participants and MI patients

	Total (n = 1224)			Controls (n = 729)			MI patients (n = 495)		
	nonMACCE (n = 1050)	MACCE (n = 174)	*P* value	nonMACCE (n = 678)	MACCE (n = 51)	*P* value	nonMACCE (n = 372)	MACCE (n = 123)	*P* value
*rs3200401 Genotype*									
C/C	755 (71.9%)	109 (62.6%)		479 (70.6%)	35 (68.6%)		276 (74.2%)	74 (60.2%)	
C/T	268 (25.5%)	62 (35.6%)		181 (26.7%)	15 (29.4%)		87 (23.4%)	47 (38.2%)	
T/T	27 (2.6%)	3 (1.7%)	0.020	18 (2.7%)	1 (2.0%)	0.885	9 (2.4%)	2 (1.6%)	0.006
*Allele*									
C	1778 (84.7%)	280 (80.5%)		1139 (84.0%)	85 (83.3%)		639 (85.9%)	195 (79.3%)	
T	322 (15.3%)	68 (19.5%)	0.047	217 (16.0%)	17 (16.7%)	0.860	105 (14.1%)	51 (20.7%)	0.014
*Dominant model*									
CC	755 (71.9%)	109 (62.6%)		479 (70.7%)	35 (68.6%)		276 (74.2%)	75 (60.5%)	
CT + TT	295 (28.1%)	65 (37.4%)	0.013	199 (29.4%)	16 (31.4%)	0.760	96 (25.8%)	49 (39.5%)	0.003
*Overdominant model*									
CT	268 (25.5%)	62 (35.6%)		181 ( 26.7%)	15 (29.4%)		87 (23.4%)	47 (38.2%)	
CC + TT	782 (74.5%)	112 (64.4%)	0.005	497 (73.3%)	36 (70.6%)	0.673	285 (76.6%)	76 (61.8%)	0.001
*Recessive model*									
TT	27 (2.6%)	3 (1.7%)		18 (2.7%)	1 (2.0%)		9 (2.4%)	2 (1.5%)	
CC + CT	1023 (97.4%)	171 (98.3%)	0.790	660 (97.4%)	50 (98.0%)	> 0.999	363 (97.6%)	130 (98.5%)	> 0.999

Similarly, in the MI patients, the MACCE group had higher frequencies of the rs3200401 T allele and CT + TT genotypes than the nonMACCE group (T allele, 20.7% vs. 14.1%, *P* = 0.014, CT + TT genotypes, 39.5% vs. 25.8%, *P* = 0.003, Table 2).

However, for the control participants, we did not observe a prominent difference in the distribution of the rs3200401 T allele or CT + TT genotypes between the MACCE group and the nonMACCE group (T allele, 16.7% vs. 16.0%, *P* = 0.860, CT + TT genotypes, 31.4% vs. 29.4%, *P* = 0.760, Table 2).

### Angiography MI patients with different *MALAT1* genotypes

Among the MI patients carrying the CT + TT genotypes or the CC genotype, there was no significant difference in the number of lesion coronary arteries (*P* = 0.381, Table 3). Meanwhile, the angiography findings, including the number of left circumflex branches, left anterior descending branch, left main, right coronary artery with lesions and revascularization, did not differ by rs3200401 genotype (*P* = 0.732, *P* = 0.076, Table 3). In addition, there was no significant difference in Gensini scores between the rs3200401 CT + TT genotypes and the CC genotype in MI patients (53 (37, 84.5) vs. 60 (35, 84), *P* = 0.567, Table 3).

**Table 3:** Angiography and revascularization of MI patients with different rs3200401 genotypes

	MI patients (n = 495)		χ^2^	*P* value
	CC genotype (n = 350)	CT + TT genotype (n = 145)		
*Lesion vessel number*				
1	94 (26.9%)	31 (21.4%)		
2	79 (22.6%)	32 (22.1%)		
≥ 3	177 (50.6%)	82 (56.6%)	1.931	0.381
*Lesion vessel*				
Left main (n, %)	30 (8.6%)	16 (11.0%)		
Left anterior descending (n, %)	300 (85.7%)	125 (86.2%)		
Left circumflex (n, %)	206 (58.9%)	91 (62.8%)		
Right coronary artery (n, %)	237 (67.7%)	115 (79.3%)	1.286	0.732
*Revascularization*				
Left main (n, %)	2 (6.7%)	4 (25.0%)		
Left anterior descending (n, %)	153 (51.0%)	55 (44.0%)		
Left circumflex (n, %)	45 (21.8%)	22 (24.2%)		
Right coronary artery (n, %)	88 (37.1%)	48 (41.7%)	6.873	0.076
GENSINI score	53 (37, 84.5)	60 (35, 84)	0.573	0.567

### MACCE risk and rs3200401 genotypes

**Fig. 1:**
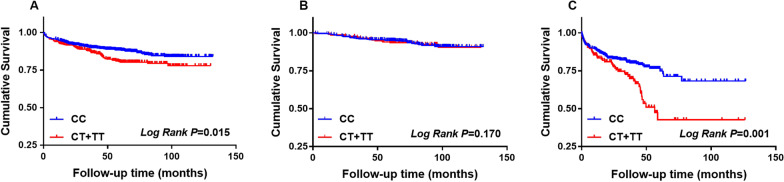
Kaplan–Meier MACCE free survival analysis according to the rs3200401 genotype. **a** in total participants. **b** in control participants. **c** in MI patients

The Kaplan–Meier analysis revealed that among all participants and MI patients, the MACCE-free cumulative survival rate in the CT + TT genotype group was obviously lower than that in the CC genotype group (*P* = 0.015, Fig. 1a, *P* = 0.001, Fig. 1c). However, in the control participants, there was no obvious difference between the CT + TT genotype group and the CC genotype group (*P* = 0.790, Fig. 1b).

Further, univariate Cox survival analysis was used to determine the risk factors for MACCEs among the total participants. The analysis showed that the rs3200401 CT + TT genotype, history of MI, male sex, age, diabetes, Gensini score, number of lesion coronary arteries, revascularization, admission blood glucose, TG, HDL-C, and ApoA were all MACCE risk factors. The multivariate Cox regression analyses suggested that the CT + TT genotypes of rs3200401 were associated with a risk for MACCEs (HR: 1.441, 95% CI 1.035–2.084, *P* = 0.031, Table 4).

**Table 4:** Univariate and multivariate Cox analyses among the total participants

Risk factors	Univariate Cox regression		Multivariate Cox regression	
	HR (95% CI)	*P* value	AHR (95% CI)	*P* value
rs3200401 CT + TT/CC	1.459 (1.073–1.984)	0.016	1.441 (1.035–2.008)	0.031
MI	6.722 (4.767–9.481)	< 0.001	3.218 (1.653–6.266)	0.001
Male	1.475 (1.071–2.024)	0.017	1.094 (0.724–1.654)	0.668
Age	1.048 (1.034–1.062)	< 0.001	1.022 (1.008–1.037)	0.002
Smoker	1.014 (0.746–1.379)	0.929	–	–
Drinker	0.856 (0.613–1.196)	0.363	–	–
Hypertension	1.299 (0.965–1.749)	0.084	–	–
Diabetes	1.799 (1.271–2.546)	< 0.001	1.072 (0.719–1.599)	0.732
BMI	0.982 (0.933–1.033)	0.476	–	–
SBP	0.996 (0.987–1.005)	0.387	–	–
DBP	0.991 (0.978–1.004)	0.169	–	–
Gensini score	1.018 (1.015–1.021)	< 0.001	1.003 (0.998–1.008)	0.271
Lesion vessel number	1.656 (1.535–1.786)	< 0.001	1.297 (1.124–1.495)	< 0.001
Revascularization	2.081 (1.763–2.457)	< 0.001	0.895 (0.656–1.22)	0.483
Glucose	1.090 (1.059–1.122)	< 0.001	1.013 (0.969–1.059)	0.567
TG	1.205 (1.033–1.404)	0.017	1.290 (1.083–1.536)	0.004
TC	1.034 (0.893–1.197)	0.657	–	–
HDL-C	0.449 (0.250–0.806)	0.007	0.647 (0.282–1.486)	0.305
LDL-C	1.171 (0.984–1.393)	0.075	–	–
ApoA	0.389 (0.205–0.738)	0.004	0.641 (0.291–1.413)	0.270
ApoB	1.148 (0.719–1.833)	0.563	–	–
Lp(a)	1.000 (0.999–1.001)	0.568	–	–

In addition, we studied the risk factors for MACCEs among MI patients with univariate Cox survival analysis. It indicated that the CT + TT genotypes of rs3200401, age, smoker, drinker, hypertension, Gensini score, number of lesion coronary arteries and TG were all risk factors for MACCE. After adjustment by multivariate Cox regression analyses, patients with the CT + TT genotypes of rs3200401 were associated with a higher risk of MACCEs than those carrying the CC genotype (HR: 1.554, 95% CI 1.060–2.277, *P* = 0.024, Table 5).

**Table 5:** Univariate and multivariate Cox analyses among the MI patients

Risk factors	Univariate Cox regression		Multivariate Cox regression	
	HR (95% CI)	*P* value	AHR (95% CI)	*P* value
rs3200401 CT + TT/CC	1.459 (1.073–1.984)	0.016	1.554 (1.060–2.277)	0.024
Male	1.018 (0.646–1.603)	0.940	–	–
Age	1.028 (1.013–1.043)	< 0.001	1.013 (0.997–1.029)	0.125
Smoker	1.605 (1.125–2.288)	0.009	1.110 (0.746–1.653)	0.608
Drinker	1.484 (1.014–2.169)	0.042	1.072 (0.715–1.608)	0.738
Hypertension	1.445 (1.015–2.059)	0.041	1.463 (1.006–2.128)	0.046
Diabetes	1.119 (0.753–1.662)	0.578	–	–
BMI	0.983 (0.923–1.047)	0.599	–	–
SBP	1.007 (0.997–1.017)	0.158	–	–
DBP	0.999 (0.985–1.013)	0.876	–	–
Gensini score	1.006 (1.001–1.011)	0.010	1.003 (0.998–1.008)	0.212
Lesion vessel number	1.353 (1.199–1.528)	< 0.001	1.299 (1.135–1.486)	< 0.001
Revascularization	0.883 (0.666–1.170)	0.387	–	–
Glucose	1.033 (0.993–1.074)	0.104	–	–
TG	1.263 (1.062–1.502)	0.008	1.242 (1.036–1.488)	0.019
TC	0.932 (0.796–1.091)	0.383	–	–
HDL-C	0.718 (0.330–1.560)	0.403	–	–
LDL-C	1.008 (0.825–1.232)	0.936	–	–
ApoA	0.838 (0.447–1.571)	0.582	–	–
ApoB	0.844 (0.459–1.551)	0.585	–	–
Lp(a)	1.000 (0.999–1.001)	0.666	–	–

## Discussion

In the present study, we investigated the relationship between the *MALAT1* gene polymorphism and MACCE and found that subjects carrying the CT + TT genotypes of rs3200401 were susceptible to MACCE, especially MI patients.

Human genome sequencing and the GENCODE project launched in 2003 have revealed that the minority of the human genome can be translated into proteins and that the majority of the genome is primarily transcribed to produce noncoding RNAs [20]. Noncoding RNAs include miRNAs, lncRNAs, and circular RNAs. To date, noncoding RNAs have been identified to regulate gene functions and shown to play a role in various biological processes, including epigenetic and transcriptional regulation [21], protein biosynthesizing processes, pluripotency and differentiation [22], embryogenesis and development [23], and dynamic developmental and cell-specific expression patterns. Currently, several lncRNAs have been reported to be involved in heart disease [5, 6, 24]. In addition, lncRNAs are also emerging as biomarkers for cardiovascular diseases [25–28]; for example, ANRIL, KCNQ1OT1, and MIAT are markers of left ventricular dysfunction in postmyocardial infarction [29].

Jian-Zhong Wang et al. first discovered that the *MALAT1* gene rs3200401 CT + TT genotypes were associated with better survival in patients with advanced lung adenocarcinoma [30]. In our study, we firstly investigated the association between the *MALAT1* rs3200401 polymorphism and MACCEs occurring among MI patients and control participants and found the rs3200401 CT + TT genotypes were independent factors of MACCEs among MI patients. Nevine Fathy et al. reported that the CT + TT genotypes of rs3200401 were the independent predictors of cerebral ischemic stroke in Egypt [31]. Moreover, Yi-Lan Li et al. found that the rs3200401 TT genotype carriers had a higher level of total cholesterol than CC + CT genotypes carriers in MI patients [32]. However, Genan Wang et al. found rs3200401 CT + TT genotypes carriers had a lower level of total cholesterol among coronary atherosclerotic heart disease patients [33]. Furthermore, it is well known that risk factors of MACCEs include age, diabetes, hypertension, dyslipidemia, and the severity of ischemia disease. Considering that the CT + TT genotypes of rs3200401 carriers had a higher concentration of total cholesterol in MI patients [32] and had a disease exacerbation among the cerebral ischemic stroke patients [31], we speculated that the lncRNA MALAT1 gene rs3200401 CT + TT genotypes may affect lipid disorders and aggravate the exacerbation in cerebral ischemia, leading to a higher incidence of MACCEs. In addition, it is reported that the rs3200401 CT + TT genotypes could be a potential genetic marker of colorectal cancer predisposition [34], and the CT + TT genotypes of rs3200401 are associated with increased risk of esophageal squamous cell carcinoma [35], while Huimin Yan found the rs3200401 CT + TT genotypes tend to elevated Parkinson’s disease susceptibility [36]. Taken together, the *MALAT1* gene rs3200401 polymorphism may be a potential functional mutation that deserves further research and may be a genetic biomarker of MACCEs susceptibility among MI patients.

In the present study, there were several limitations. First, this was not a multicenter and large-sample study, which would provide less robust statistical estimations. Second, compared with the MACCE group, the participants were a little older than the participants in the nonMACCE group and it may leave the possibility that the predictive power of the MALAT1 gene rs3200401 polymorphism for MACCE could be influenced. After adjusting the confounders via multivariate Cox regression analyses, the MALAT1 gene rs3200401 CT + TT genotypes remained the risk factor for MACCEs in MI patients. However, age was not the risk factor for MACCEs so the age was not the confounder of MACCEs in this study which did not significantly influence the power of MALAT1 gene rs3200401 polymorphism for MACCEs. Third, we only analyzed the association between *MALAT1* gene rs3200401 CT + TT genotypes and MACCE. However, we did not measure the level of lncRNA MALAT1 in plasma, resulting in the linkage between the expression of lncRNA MALAT1 in plasma and MACCE being undetected, which deserves further research.

In summary, these results indicated that the *MALAT1* gene played an important role in the prognosis of MI patients and that the *MALAT1* gene rs3200401 CT + TT genotypes are risk factors for the poor prognosis of MI patients, suggesting that the *MALAT1* gene can be used as a biomarker susceptible to MACCE for MI patients. Further gene sequencing for individuals with traditional risk factors could help to identify whether they carry *MALAT1* rs3200401 CT + TT genotypes, which may help clinicians distinguish high-risk MACCE patients with a history of MI. This is also the real purpose of precision medicine.

## Conclusions

In conclusion, our study revealed that *MALAT1* rs3200401 CT + TT genotypes could be a risk factor for MACCEs in MI patients, suggesting that the *MALAT1* gene may become a biomarker for poor prognosis in MI patients.

## Data Availability

The datasets generated and analyzed during the current study are available in the NCBI dbSNP database, SUB11120626.
